# The Physiology Constant Database of Teen-Agers in Beijing

**DOI:** 10.1100/tsw.2004.98

**Published:** 2004-06-14

**Authors:** Wei Wei-Qi, Zhu Guang-Jin, Xu Cheng-Li, Han Shao-Mei, Qi Bao-Shen, Chen Li, Zu Shu-Yu, Zhou Xiao-Mei, Hu Wen-Feng, Zhang Zheng-Guo

**Affiliations:** ^1^ Department of Biomedical Engineering, Institute of Basic Medical Sciences, Peking Union Medical College, Chinese Academy of Medical Sciences, China; ^2^ Department of Pathophysiology, Institute of Basic Medical Sciences, Peking Union Medical College, Chinese Academy of Medical Sciences, China; ^3^ Department of Physiology, Institute of Basic Medical Sciences, Peking Union Medical College, Chinese Academy of Medical Sciences, China; ^4^ Department of Epidemiology and Biostatistics, Institute of Basic Medical Sciences, Peking Union Medical College, Chinese Academy of Medical Sciences, China; ^5^ Instrument and techniques, Institute of Basic Medical Sciences, Peking Union Medical College, Chinese Academy of Medical Sciences, China

**Keywords:** human development, adolescents, physiology, public health, China

## Abstract

Physiology constants of adolescents are important to understand growing living systems and are a useful reference in clinical and epidemiological research. Until recently, physiology constants were not available in China and therefore most physiologists, physicians, and nutritionists had to use data from abroad for reference. However, the very difference between the Eastern and Western races casts doubt on the usefulness of overseas data. We have therefore created a database system to provide a repository for the storage of physiology constants of teen-agers in Beijing. The several thousands of pieces of data are now divided into hematological biochemistry, lung function, and cardiac function with all data manually checked before being transferred into the database. The database was accomplished through the development of a web interface, scripts, and a relational database. The physiology data were integrated into the relational database system to provide flexible facilities by using combinations of various terms and parameters. A web browser interface was designed for the users to facilitate their searching. The database is available on the web. The statistical table, scatter diagram, and histogram of the data are available for both anonym and user according to queries, while only the user can achieve detail, including download data and advanced search.

## INTRODUCTION

Physiology constants of teen-agers are important for the understanding of growing living systems. According to these data, a constant range of teen-age body conditions could be established. With the constants as important guides and references, it will be easier for clinicians to distinguish the different body conditions. The constant data also reflect the public physiological variance of different time and areas. Until recently, there was no consummate physiology constants work done in China. Historically, most physiologists, physicians, and nutritionists had to use data from abroad for reference. However, the very difference between the Eastern and Western races casts doubt about the value of overseas data[1,2,3,4,5,6]. Living conditions and quality changing are different and some of the data from medical sources do not represent the current condition[1]. This lack of data has made it imperative to create a teen-age physiology constant survey for China.

## METHODS

Based on the population proportion and economic level, three districts were chosen from the eighteen districts of Beijing. In each district, the school was taken as the first stage-sampling unit and chosen randomly. The grade and class were taken as the second and the last stage-sampling unit. This sampling method was helpful to guarantee the generalization of samples and a larger dataset of each unit. Since the health survey of Chinese students in 1985 and 1995[7] indicated that there was physical development variance between students in the city and the countryside[1], we divided Beijing as the main sampling layer into two sublayers, city layer and countryside layer. In each sublayer, there were elementary school, primary school, and senior high school layers.

Because of the amount of data, it became important to assemble them in an efficient way and to disseminate them for usage. We divided those data into hematological biochemistry, lung function, or cardiac function and developed a database system. All data were dual-inputted before being transferred into the database. This double manual check should keep the data reliable.

The database was implemented as a B/S (Browser/Server) structure. In this project, Mysql 4.0 was chosen as the database server software, since it is an open source database with high performance and reliability.

The database relies on a relational structure. There are four tables in it, one for demography data, one for hematological biochemistry, one for lung function (respiration system), and one for cardiac function. Since all of them are related to demographic data, an individual demography has been created to avoid redundant data structure and repeated entries. All those tables are linked through “ID”, the primary key (see Table 1).

## RESULTS

The purpose of our database is to store and offer simple tools to analyze the physiology constants data of Beijing teen-agers. Now the database has become available on the World Wide Web (http://168.160.62.35/CNHPC/). The database users are those who are studying physiology or subjects related to it. The statistical table, scatter diagram, and histogram of the data are available for both anonym and user according to queries, while only the user can achieve detail, including download data and advanced search. The interested people can write to the author for a guest username and the password.

There are two ways to query in the database: normal and advanced search. In the normal search, the user has to choose in which database he/she wants to perform the query, like hematological biochemistry, lung function, or cardiac function. Then the user specifies a query by filling in a form. For example, a query page of hematological biochemistry tests is shown in Fig. 1. According to their purposes and conveniences, the user can search by up to seven conditions, like sex, age, weight, and height. The user

**TABLE 1**
**The Abbreviated Description of Tables in Database with an Example Entry (Underlines Indicate the Primary Keys and Hyperlinks to Details)**

| **Hematological Biochemistry** | | **Demographic Data** | |
|---|---|---|---|
| ID | 12309 | ID | 12309 |
| BMI | 20.40 | AGE | 11 |
| TP | 7.60 | SEX | 1 |
| ALB | 4.60 | T4 (Height) | 149.0 |
| ALT | 25.00 | T5 (Weight) | 46.0 |
| GLU | 87.00 | | |
| Ca | 9.30 | | |
| P | 5.20 | | |
| A_G | 1.50 | | |
| **Lung Function (Respiration System)** | | **Cardiac Function** | |
| ID | 12309 | ID | 12309 |
| VT | 0.41 | HR | 0 |
| BF | 23.76 | SBP | 0 |
| MV | 9.76 | DBP | 0 |
| ERV | 0.62 | MAP | 0 |
| IC | 1.97 | CI | 5.1 |
| VC | 2.59 | CO | 9.5 |
| FVC | 2.32 | SV | 128 |
| FEV1 | 2.11 | SVR | 0 |
| FEV1_FVC | 90.94 | LCW | 0 |
| PEF | 4.08 | LVET | 0.31 |
| PEF25 | 3.58 | | |
| PEF50 | 2.20 | | |
| PEF75 | 0.93 | | |
| MMEF75_25 | 1.94 | | |
| MVV | 56.15 | | |

can also restrict the output options at the right side of the table. After this, the program will check to see if all data are entered correctly (e.g., if user's condition is "age between 12 and 16", the latter value should be larger than or equal to the former one). If all conditions have been written correctly, the results are returned as hypertext allowing the user to navigate to related information.

The advanced search supports the query among databases (see Fig. 2). For example, if the user wants to know the BMI values in the hematological biochemistry system and the VT values in the respiratory system between 0.1 and 0.3, there is no chance to get the results by normal search. However, through the advanced search, it will be easy to realize. The user may follow the form and fill the different conditions; the data will be combined and displayed.

Retrieved data are shown in Fig. 3. Since the result table might be quite long, each page will return no more than 50 results. A sorting function is available and the user can access the detailed information through the ID linkage. The ID link will search all the hematological biochemistry, lung function, and cardiac function tests. The detailed information will be returned if it exists. Automated statistical program is implemented. Maximum, minimum, average, and standard deviation are displayed by different colors.

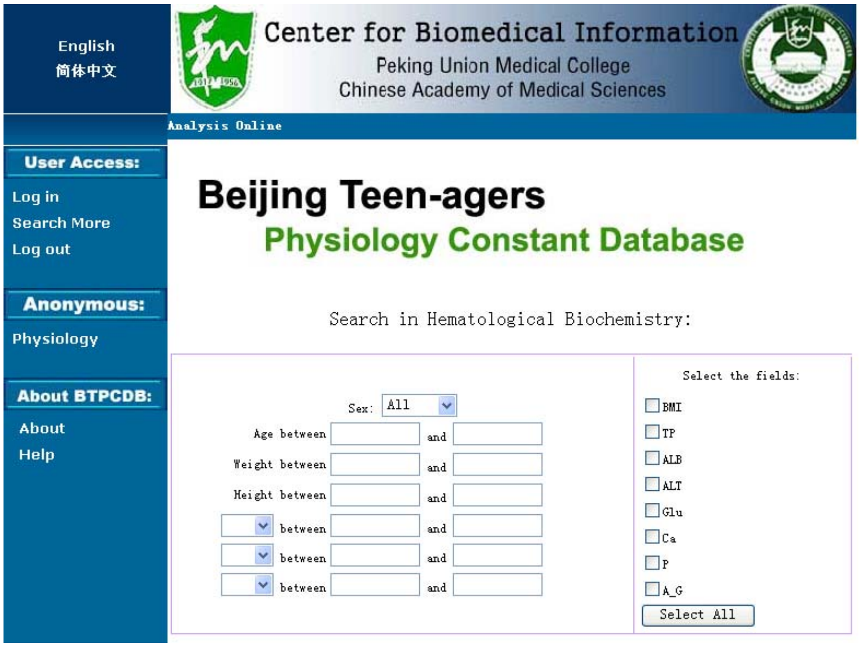

**FIGURE 1**. Hematological biochemistry query interface.

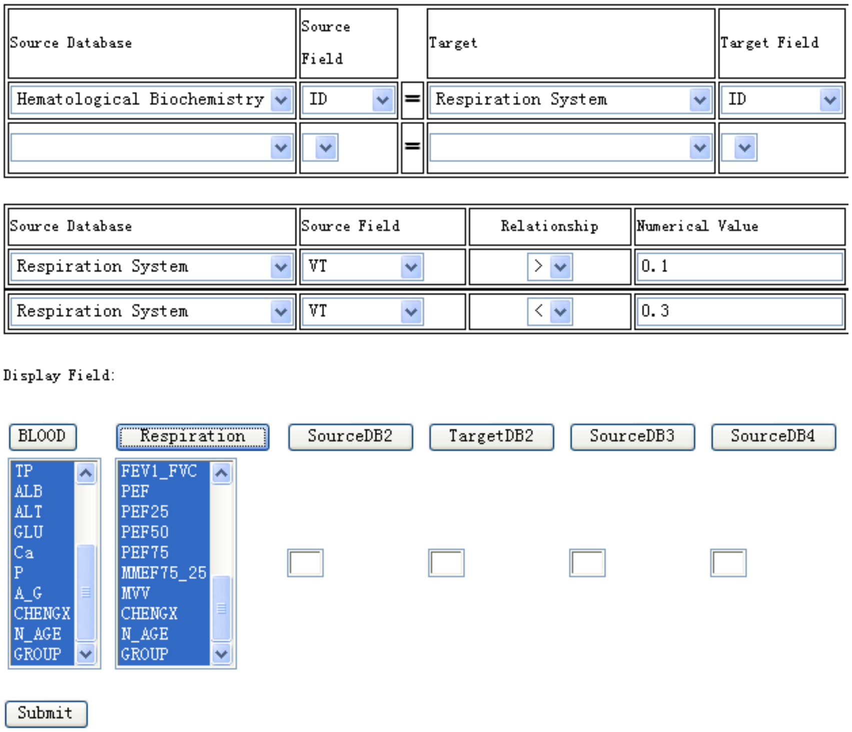

**FIGURE 2**. Advanced search.

The condition is sex:male
Total:1441|The first 50 pieces are shown.download data here!

| | Statistics \|Average\|Standard Deviation | | Statistics \|Average\|Standard Deviation |
|---|---|---|---|
| Age | 9-29\|14.6988\|3.1620 | Sex | Male:1441 \|(100%)\|Female:0(0%) \|Total:1441 |
| Weight | 26.2-125.0\|54.40625\|14.43518 | Height | 100.4-191.0\|162.11874\|12.95580 |
| BMI | 13.30-43.30\|20.381131\|3.469413 | TP | 5.80-9.10 \|7.325468 \|0.435671 |
| ALB | 3.70-5.50 \|4.529493 \|0.239616 | ALT | 5.00-230.00 \|16.985357 \|13.249125 |
| GLU | 58.00-152.00 \|84.236641 \|10.217764 | Ca | 8.00-11.30 \|9.634976 \|0.562658 |
| P | 1.50-6.00 \|4.331159 \|0.609883 | A_G | 1.00-3.60 \|1.651561 \|0.245570 |

Page 1 2 3 4 5 6 7 8 9 10 11 12 13 14 15 16 17 18 19 20 21 22 23 24 25 26 27 28 29

| ID | Age | Sex | Weight(Kg) | Height(cm) | BMI | TP | ALB | ALT | Glu | Ca | P | A_G |
|---|---|---|---|---|---|---|---|---|---|---|---|---|
| 11103 | 12 | M | 45.0 | 148.5 | 20.40 | 7.60 | 4.60 | 25.00 | 87.00 | 9.30 | 5.20 | 1.50 |
| 11126 | 13 | M | 40.5 | 151.0 | 17.80 | 7.30 | 4.60 | 11.00 | 92.00 | 9.20 | 5.70 | 1.70 |
| 11132 | 12 | M | 44.4 | 156.0 | 18.20 | 6.90 | 4.40 | 6.00 | 68.00 | 8.80 | 5.50 | 1.80 |
| 11143 | 13 | M | 42.4 | 145.5 | 20.00 | 6.90 | 4.10 | 13.00 | 93.00 | 8.90 | 4.60 | 1.50 |

**FIGURE 3**. One user’s query result.

## DATA DOWNLOAD

Some researchers are not content to look up data. At our institute, most researchers will use all kinds of data for analysis by other statistical programs, like SAS, SPSS. The database offers a download function that generates a text data file according to the user’s query condition. The downloaded data file could be opened by most commercial software for further analysis.

## SCATTER PLOT AND HISTOGRAM

There are two on-line graphics tools available: scatter plot and histogram. In the scatter plot, the user may specify the x-axis, y-axis, limited conditions, and determinate whether a linear regression is necessary. As the result, the scatter plot will reflect the relationship between the chosen properties (see Fig. 4).

Histograms will be useful to check the distribution of data. In this database, we offer an on-line histogram program to draw one histogram that can reflect distributions of up to four different conditions. Through the different colors, the distributions will be easy to contrast and evaluate.

## DISCUSSION

The teen-agers’ physiology constants are indicators of growing living systems. Studying the growing trends of teen-agers offers us a preferential condition to prevent adult health problems. The accumulation of teen-agers’ physiology constants is also important for labor and public health departments to constitute proper standards and the normal range for the certain area and race. This project collects thousands of physiology constants of current teen-agers. It reflects the real teen-age situation in Beijing. Clinicians can use the result as main references to diagnose diseases and physiologists and nutritionists can use it for research work.

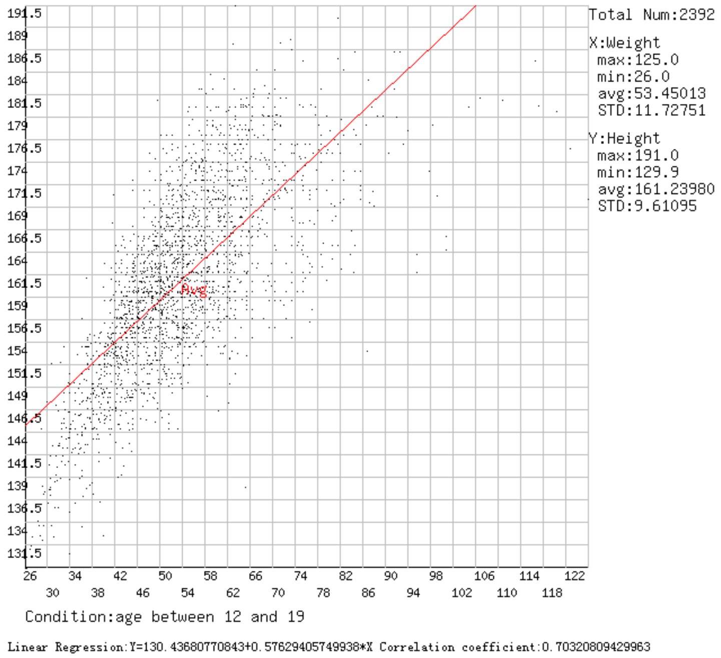

**FIGURE 4**. One scatter plot result.

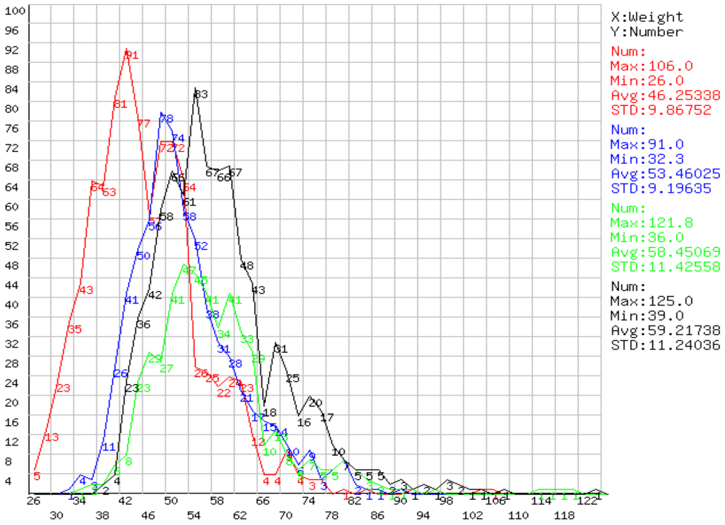

**FIGURE 5**. One histogram.

The physiology constants are changing data according to races, areas, economic levels, and dietetic customs. We recommend that the database should be updated in a limited time. In addition, more physiology constants that reflect other organs (e.g., liver, kidney) should be added.

When data accumulate to thousands there will be a problem of how to clean the data. Here, we used the double manual check, but it caused a lot of additional effort and cost. A well-planned survey procedure might be helpful to lessen it. Upgrading will be unavoidable, no matter how carefully the system is designed. In this project, we had to revise several times, since the requirement was changing and the understanding improved. The only way to reduce this risk is to plan for it. There is no perfect system, but we can steadily improve it.

Communication is very important. In our project, most of us had graduated from medical school and we therefore understood each other's questions and needs better. In our experience, communication was invaluable.

## ACKNOWLEDGMENTS

This project was supported by China Medical Board of New York, Inc. (#03-787), National Natural Science Foundation of China (#30370394), Ministry of Science and Technology (#2001DEA30031), and Ministry of Science and Technology (#2001DEA30032).

## BIOSKETCHES

**Wei Wei-Qi, MS** candidate in the Biomedical Engineering Department at the Chinese Academy of Medical Sciences and Peking Union Medical College, 100005 P.R. China. His major interests include medical informatics and biomedical engineering. E-mail: wqwei@pumc.edu.cn

**Zhang Zheng-Guo,** Medical Master Degree, Director of Department of Biomedical Engineering, Peking Union Medical College, Chinese Academy of Medical Sciences, 100005 P.R. China. E-mail: z.g.zhang@ieee.org
